# Neoadjuvant Toripalimab Plus Axitinib for Nonmetastatic Clear Cell Renal Cell Carcinoma With Tumor Thrombus: A Combined Analysis of Two Phase II Clinical Trials

**DOI:** 10.1002/mco2.70720

**Published:** 2026-04-06

**Authors:** Cheng Peng, Zhuolong Wu, Yaohui Wang, Qiyang Liang, Dongxing Wang, Houming Zhao, Jinhang Li, Xiuzheng Yue, Yibo Zhang, Jialong Song, Changwei Shi, Haiyi Wang, Guoqiang Yang, Baojun Wang, Qingbo Huang, Xu Zhang, Xin Ma, Jiwei Huang, Liangyou Gu

**Affiliations:** ^1^ Senior Department of Urology Chinese PLA General Hospital Beijing China; ^2^ Department of Urology School of Medicine RenJi Hospital Shanghai Jiao Tong University Shanghai China; ^3^ Chinese PLA Medical School Chinese PLA General Hospital Beijing China; ^4^ Department of Pathology The First Medical Center Chinese PLA General Hospital Beijing China; ^5^ Medical Big Data Research Center Medical Innovation Research Division of PLA General Hospital Beijing China; ^6^ School of Biomedical Engineering Wenzhou Medical University Wenzhou China; ^7^ School of Medicine Nankai University Tianjin China; ^8^ Department of Radiology The First Medical Center Chinese PLA General Hospital Beijing China

**Keywords:** drug resistance, neoadjuvant immunotherapy‐based combination, renal cell carcinoma, thrombectomy, venous tumor thrombus

## Abstract

Neoadjuvant immunotherapy‐based combination holds promises in reducing surgical risk and improving survival for renal cell carcinoma (RCC) with venous tumor thrombus (VTT). However, its role in RCC–VTT has been less explored. To evaluate the efficacy and safety of neoadjuvant toripalimab plus axitinib in nonmetastatic RCC–VTT, we conducted a combined analysis of two Phase II trials with similar design. Thirty‐four patients with nonmetastatic clear cell RCC (ccRCC) and Mayo Level 0–IV VTT were enrolled. Toripalimab plus axitinib was administered for up to 12 weeks before surgery. The primary endpoint was objective response rate (ORR). In this study, the ORR and disease control rates were 41% (14 out of 34) and 97% (33 out of 34), respectively. 47% (16 out of 34) patients experienced a reduction in VTT levels. Grade 3 treatment‐related adverse events (TRAEs) occurred in 24% (eight out of 34) patients, and no Grade 4 or 5 TRAEs were observed. Thirty patients were eligible for surgery, and the surgical strategy was simplified in 53% (16 out of 30) patients. One‐year disease‐free survival and overall survival were 76.7% (95% CI, 59.1–88.2%) and 91.2% (95% CI, 77.0–97.0%), respectively. Multiomics analysis revealed the nonresponder group exhibited significant tumor heterogeneity and a stroma‐characterized tumor microenvironment. In conclusion, neoadjuvant toripalimab plus axitinib was clinically active and safe in patients with nonmetastatic ccRCC–VTT.

## Graphical abstract

This combined analysis demonstrates that neoadjuvant toripalimab plus axitinib is clinically active and safe for nonmetastatic RCC with venous tumor thrombus. Exploratory multiomics profiling linked treatment resistance to a distinct tumor microenvironment characterized by heightened heterogeneity and stromal activity, providing a new strategy for the perioperative management and novel biological insights for this high‐risk disease.

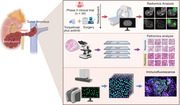

## 1 Introduction

Renal cell carcinoma (RCC) is frequently characterized by the invasion of the renal hilar vasculature, leading to the formation of a venous tumor thrombus (VTT), which is a hallmark of locally advanced disease [1, 2]. Tumor thrombectomy remains the standard treatment for patients with nonmetastatic disease, with a surgical strategy guided by the Mayo Clinic classification system [2]. However, this procedure demonstrates high rates of perioperative complications (12–47%) and mortality (5–15%), which increase with the levels of VTT [3, 4]. Thus, there is a noteworthy necessity to increase surgical safety and improve oncological outcomes for this high‐risk group.

Neoadjuvant therapy aims to reduce tumor burden before surgical debulking and improve operative and survival outcomes [5, 6]. Patients with VTT may benefit from neoadjuvant therapy by downstaging tumor thrombus and reducing surgical complexity. Although many retrospective studies have confirmed significant reduction in VTT height after neoadjuvant tyrosine kinase inhibitor (TKI) monotherapy, the limited downstaging rate of thrombus levels (<30%) restricts its potential as a standard preoperative treatment [7, 8]. Recently, the combinations of TKIs and immune checkpoint inhibitors (ICIs) have achieved a substantial improvement in life expectancy with significantly improved objective response rate (ORR) up to 59–71% in advanced clear cell RCC (ccRCC) [9, 10], revolutionizing the treatment landscape. It has driven a growing interest of neoadjuvant immunotherapy‐based combination (IBC) therapeutic strategies for RCC.

To date, the effect of IBC in the neoadjuvant setting for RCC–VTT has been less explored and no standard neoadjuvant therapies have been approved for locally advanced RCC. Our prior study (NEOTAX trial) represents the first Phase II investigation to evaluate neoadjuvant IBC treatment in the management of RCC with VTT. This study demonstrated clinically meaningful efficacy, with a downstaging rate of 44%, an ORR of 40%, and an acceptable safety profile [11]. However, this study has several limitations, including single center with small sample size, enrollment restricted to Mayo Level II–IV tumor thrombus and inclusion of metastatic cases. To overcome those limitations, we conducted a combined analysis of two reported single‐arm pilot trials [11, 12] with a similar target population and design. In this study, we included patients with ccRCC–VTT from two centers: the Chinese PLA General Hospital (PLAGH) cohort and the Renji cohort. In order to minimize intercohort heterogeneity, we excluded patients with metastatic disease.

Our study aimed to further evaluate the efficacy and safety of neoadjuvant toripalimab plus axitinib in a larger multicenter cohort of patients with nonmetastatic ccRCC–VTT (Mayo Level 0–IV), thereby providing new evidence for neoadjuvant IBC in VTT management. Additionally, we conducted multiomics and subgroup analyses to identify potential clinicopathological and radiological features associated with response to neoadjuvant IBC treatment. This combined analysis demonstrated that neoadjuvant toripalimab plus axitinib showed a high activity in downstaging the thrombus level and simplifying surgical complexity. Multiomics analysis revealed that the nonresponder group exhibited significant tumor heterogeneity and stroma‐characterized tumor microenvironment. Overall, the neoadjuvant IBC treatment provides a new strategy for the perioperative management of ccRCC with VTT.

## 2 Results

### 2.1 Characteristics of Patients and Treatment

**FIGURE 1:**
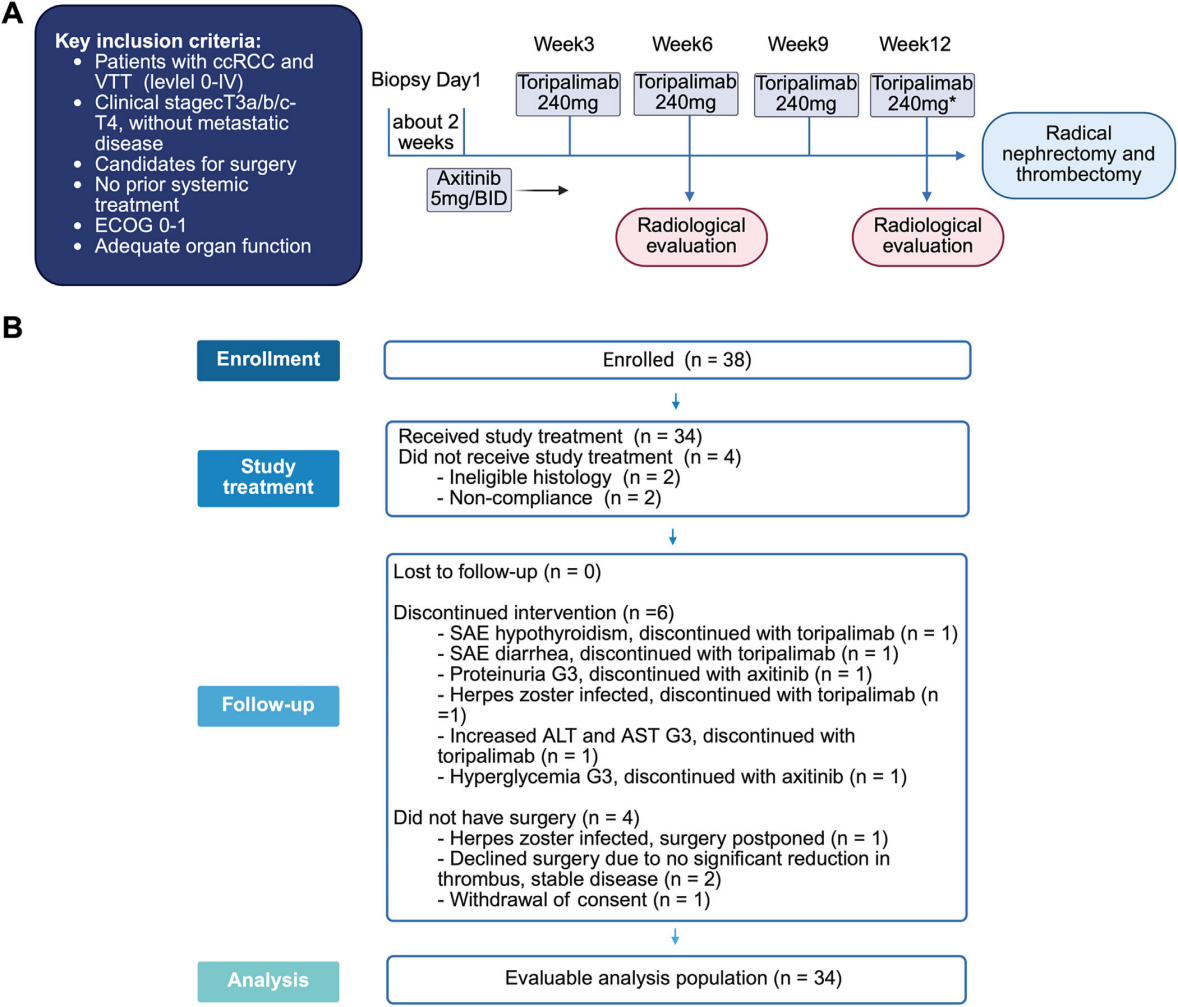
Study diagram of the two trials and the overall flowchart. (A) Study design. *The NEOTAX trial administered toripalimab as neoadjuvant therapy for four cycles, whereas the NCT04118855 trial maintained a maximum of three cycles. Description and the difference comparison of the statistical designs and inclusion/exclusion criteria between the two trials were shown in Table S5. (B) Flow diagram.

The flow chart of this study is shown in Figure 1. In this pooled analysis, 34 patients were enrolled from the two trials, of which 28 (82%) were male. The median age was 60 years (interquartile range [IQR]: 54–68 years). Regional lymph node metastases were observed in eight (24%) patients at enrollment. Three (9%), four (12%), 12 (35%), six (18%), and nine (26%) patients had Level 0, I, II, III, and IV tumor thrombi at baseline, respectively (Table 1).

**TABLE 1:** Baseline demographic and clinical characteristics of patients.

Variable	Overall (*N* = 34)	PLAGH cohort (*N* = 20)	Renji cohort (*N* = 14)	*p* Value
Median age, year (IQR)	60 (54–68)	58 (52–68)	63 (54–71)	0.599
Sex, *n* (%)				0.364
Male	28 (82)	15 (75)	13 (93)	
Female	6 (18)	5 (25)	1 (7)	
Median BMI, kg/m^2^ (IQR)	23.2 (20.6–25.3)	24.1 (21.5–26.2)	23.3 (19.6–25.5)	0.327
ECOG performance status, *n* (%)				0.627
0	28 (82)	17 (85)	11 (79)	
1	6 (18)	3 (15)	3 (21)	
Tumor laterality, *n* (%)				1.000
Right	23 (68)	14 (70)	9 (64)	
Left	11 (32)	6 (30)	5 (36)	
Clinical T stage, *n* (%)				0.013
T3a	4 (12)	0 (0)	4 (29)	
T3b	20 (59)	12 (60)	8 (57)	
T3c	9 (26)	7 (35)	2 (14)	
T4	1 (3)	1 (5)	0 (0)	
Clinical N stage, *n* (%)				0.102
N0	26 (76)	13 (65)	13 (93)	
N1	8 (24)	7 (35)	1 (7)	
Primary tumor size, cm (IQR)	8.2 (6.8–10.7)	8.2 (7.0–10.6)	8.6 (6.6–11.7)	0.624
Length of TT, cm (IQR)	11.4 (8.4–15.0)	12.4 (9.8–15.9)	9.1 (6.4–13.7)	0.234
Histological subtype on baseline biopsy, *n* (%)				
ccRCC	34 (100)	20 (100)	14 (100)	1.000
Mayo level of TT at baseline				<0.001
0	3 (9)	0 (0)	3 (21)	
I	4 (12)	0 (0)	4 (29)	
II	12 (35)	7 (35)	5 (36)	
III	6 (18)	6 (30)	0 (0)	
IV	9 (26)	7 (35)	2 (14)	

Abbreviations: BMI, body mass index; ccRCC, clear cell renal cell carcinoma; ECOG, Eastern Cooperative Oncology Group; IQR, interquartile range; TT, tumor thrombus.

### 2.2 Treatment Efficacy

**FIGURE 2:**
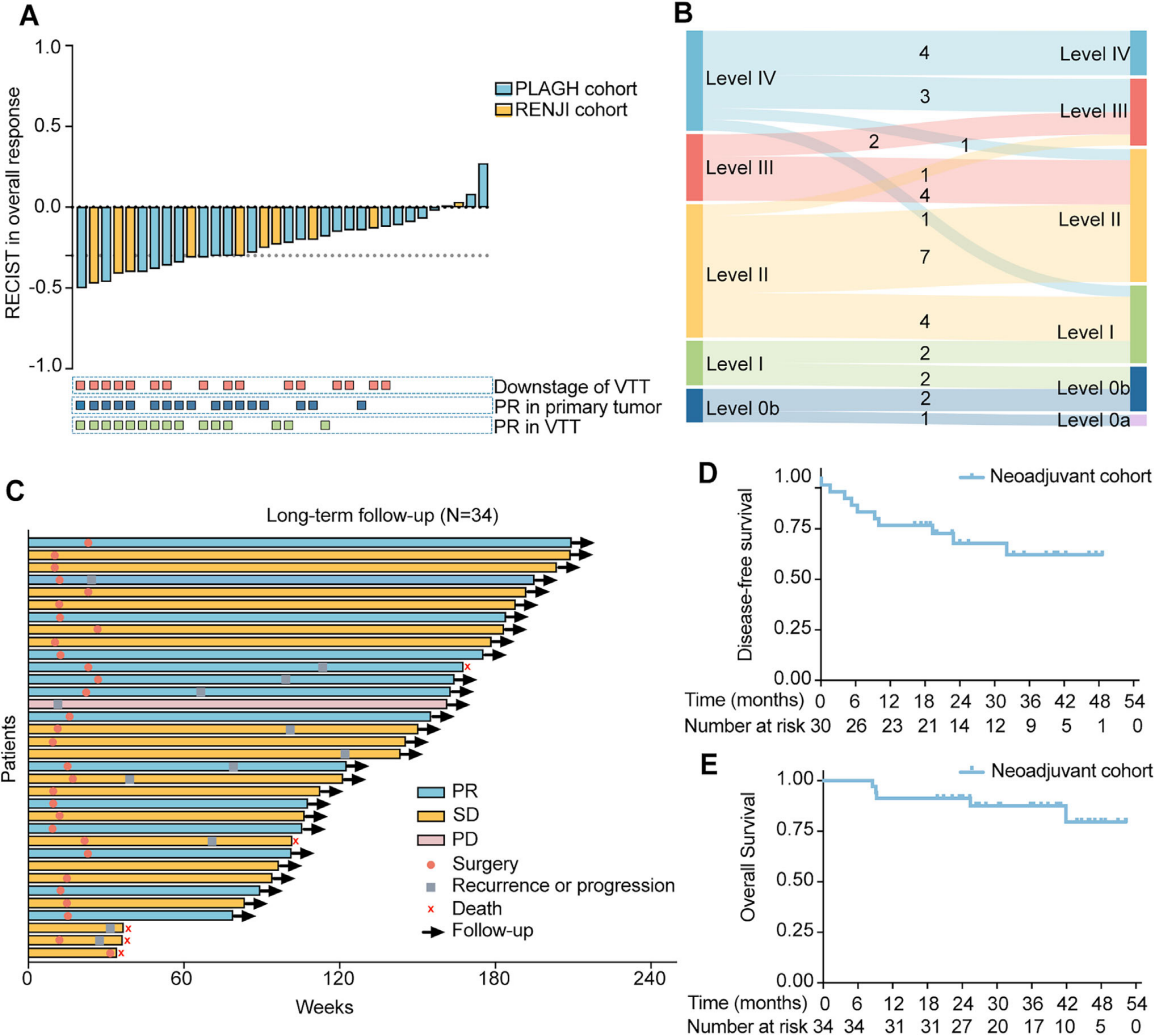
Treatment response and survival analysis of the two Phase II trials. (A) Waterfall plot of the overall response (primary tumor and venous tumor thrombus, RECIST V1.1). Concurrently, the downstage and partial response in tumor thrombus or primary tumor were also presented at bottom. (B) Sankey diagram showed the changes of TT level. Ultimately, 47% (16 out of 34) patients experienced a reduction in TT level. (C) Swimmer plot of all patients from the start of treatment to surgery, disease progression or death. (D) Kaplan–Meier plot of disease‐free survival. (E) Kaplan–Meier plot of overall survival. PD, progressive disease; PR, partial response; SD, stable disease; TT, tumor thrombus.

After 12 weeks of treatment, according to the Response Evaluation Criteria in Solid Tumors (RECIST) criteria, 41% (14 out of 34) patients achieved the best overall ORR, and the overall disease control rate (DCR) was 97% (Table 2). Radiological response assessment of the VTT and primary tumor (PT) showed a 44% ORR for VTT and 50% ORR for PT (Table 2 and Figure 2A). Two (6%) patients experienced disease progression of VTT.

**TABLE 2:** Tumor response.

Variable	Overall (*N* = 34)	PLAGH cohort (*N* = 20)	Renji cohort (*N* = 14)	*p* Value
Overall response, *n* (%)				0.580
Complete response	0 (0)	0 (0)	0 (0)	
Partial response	14 (41)	9 (45)	5 (36)	
Stable disease	19 (56)	11 (55)	8 (57)	
Progressive disease	1 (3)	0 (0)	1 (7)	
DCR, *n* (%)	33 (97)	20 (100)	13 (93)	0.412
Response by RECIST criteria in tumor thrombus, *n* (%)				0.746
PR	15 (44)	10 (50)	5 (36)	
SD	17 (50)	9 (45)	8 (57)	
PD	2 (6)	1 (5)	1 (7)	
Response by RECIST criteria in primary tumor, *n* (%)				0.728
PR	17 (50)	9 (45)	8 (57)	
SD	17 (50)	11 (55)	6 (43)	
PD	0 (0)	0(0)	0 (0)	
Change in thrombus level, *n* (%)				0.586
Increased	1 (3)	0(0)	1 (7)	
Stable	17 (50)	10 (50)	7 (50)	
Decreased	16 (47)	10 (50)	6 (43)	
Reduction in thrombus length, cm; median (IQR)	−2.4 (−4.0 to −0.8)	−3.0 (−4.0 to −1.5)	−1.1 (−3.0 to 0.1)	0.142
Reduction in primary tumor size, cm; median (IQR)	−2.0 (−3.3 to −0.7)	−1.6 (−2.8 to −0.6)	−2.1 (−3.8 to −1.3)	0.270
Median DFS, months(95% confidence interval)	NE	32.1 (0.0–64.8)	NE	0.048
12‐month DFS rate, %	76.7 (59.1–88.2)	64.7 (41.3–82.7)	92.3 (66.7–99.6)	0.104
Median OS, months(95% confidence interval)	NE	NE	NE	0.025
12‐month OS rate, %	91.2 (77.0–97.0)	85.0 (64.0–94.8)	100.0 (78.5–100.0)	0.251
Median OS follow‐up time, months(IQR)	30.2 (25.0–44.9)	27.8 (21.8–40.9)	42.5 (27.8–47.4)	0.019

Abbreviations: DFS, disease‐free survival; IQR, interquartile range; NE, not estimable; OS, overall survival. PR, partial response, SD, stable disease, PD, progressive disease; RECIST, Response Evaluation Criteria in Solid Tumors; TT, tumor thrombus.

47% (16 out of 34) patients achieved a reduction in VTT levels (IV–III in three patients, IV–II in one patient, IV–I in one patient, III–II in four patients, II–I in four patients, I–0 in two patients and downstaging of Level 0 in one patient). Only one patient (3%) showed an increase in Mayo Level (II–III). Thirty‐one (91%) patients exhibited shrinkage in VTT length, with a median reduction of 2.4 cm (IQR: 0.8–4.0 cm).

### 2.3 Perioperative Outcomes

Thirty patients who were eligible for surgery underwent radical nephrectomy with tumor thrombectomy. One patient (3%) had a 3‐week delay in surgery after 12 weeks of treatment due to Grade 3 hyperglycemia and another patient (3%) required a surgery postponement due to herpes zoster infection and poor general condition. One patient (3%) declined surgery due to disease progression during treatment, while two patients (6%) with Level IV tumor thrombus refused surgery due to limited therapeutic benefit (stable disease [SD]) and high surgical risk. Fourteen (47%) patients underwent minimally invasive surgeries, and 16 (53%) patients underwent open surgeries (Table 3). In 53% (16 out of 30) of patients, the surgical strategy changed from initial plans (avoiding cardiac arrest and extracorporeal circulation or liver mobilization and other complex procedures). The median operative time was 260 min (IQR: 180–326 min). The median estimated blood loss was 550 mL (IQR: 300–1425 mL). The rate of conversion to open surgery was 7% (one out of 15). The postoperative complication rate was 63% (19 out of 30), common complications included anemia, hypoalbuminemia, and acute kidney injury. Four patients (13%) experienced major complications, including two patients developed severe renal insufficiency requiring dialysis, one patient experienced cardiac arrest and recovered well after external cardiac compression, and one patient died due to severe hemorrhage leading to disseminated intravascular coagulation. None of the major complications were related to axitinib or toripalimab.

**TABLE 3:** Perioperative outcomes.

Variable	Overall (*N* = 30)	PLAGH cohort (*N* = 17)	Renji cohort (*N* = 13)	*p* Value
Surgical approach, *n* (%)				<0.001
Open	16 (53)	4 (24)	12 (92)	
Laparoscopic	1 (3)	0 (0)	1 (8)	
Robotic	13 (43)	13 (76)	0 (0)	
Surgery type, *n* (%)				0.024
Thrombectomy	24 (80)	11 (65)	13 (100)	
Cavectomy	6 (20)	6 (35)	0 (0)	
Operative time (min), median (IQR)	260 (180–326)	310 (203–458)	215 (155–300)	0.048
Estimated blood loss (mL), median (IQR)	550 (300–1425)	700 (300–2000)	450 (250–1050)	0.385
Intro‐operative blood transfusion				
No. pts (%)	15 (50)	9 (53)	6 (46)	1.000
Median ml (IQR)	270 (0–1200)	540 (0–1130)	0 (0–1200)	0.982
ICU, *n* (%)	17 (57)	9 (53)	8 (62)	0.721
ICU stay (d), median (IQR)	2 (0–3)	2 (0–3)	1 (0–3)	0.390
Median days (IQR)				
To surgical drain removal	4 (3–8)	5 (4–8)	3 (3–7)	0.134
To full ambulation	1 (1–3)	1 (1–3)	1 (1–3)	0.875
To oral feeding	2 (1–3)	2 (1–3)	2 (1–3)	0.774
Postoperative hospital stays (d), median (IQR)	7 (6–10)	7 (6–11)	6 (4–9)	0.169
Conversion to open, *n* (%)	1 (7)	0 (0)	1 (50)	0.433
Postoperative complications, *n* (%)	19 (63)	10 (59)	9 (69)	0.708
Clavien‐Dindo Grade, *n* (%)				0.666
Grade I	4 (13)	1 (6)	3 (23)	
Grade II	11 (37)	6 (35)	5 (38)	
Grade III	0 (0)	0 (0)	0 (0)	
Grade IV	3 (10)	2 (12)	1 (8)	
Grade V	1 (3)	1 (6)	0 (0)	

Abbreviations: ICU, intensive care unit; IQR, interquartile range.

### 2.4 Safety and Tolerability

All patients (100%, 34 out of 34) experienced at least one treatment‐related adverse event (TRAE) (Table S1), with the majority classified as Grade 1 or 2 events (76%, 26 out of 34). The most common TRAEs (of any grade) were hypertension (32%), proteinuria (29%), fatigue (29%), and diarrhea (29%). Grade 3 TRAEs were reported in 24% (eight out of 34) patients and none of the patients experienced Grade 4 or 5 TRAEs. Six (18%) patients required permanent treatment discontinuation due to Grade 3 TRAEs. This included two patients who discontinued axitinib (due to proteinuria and hyperglycemia), three patients who discontinued toripalimab (due to hypothyroidism, diarrhea, and liver dysfunction), and one patient who discontinued toripalimab and postponed surgery following herpes zoster infection.

### 2.5 Association of Multiomics Features with Treatment Response

**FIGURE 3:**
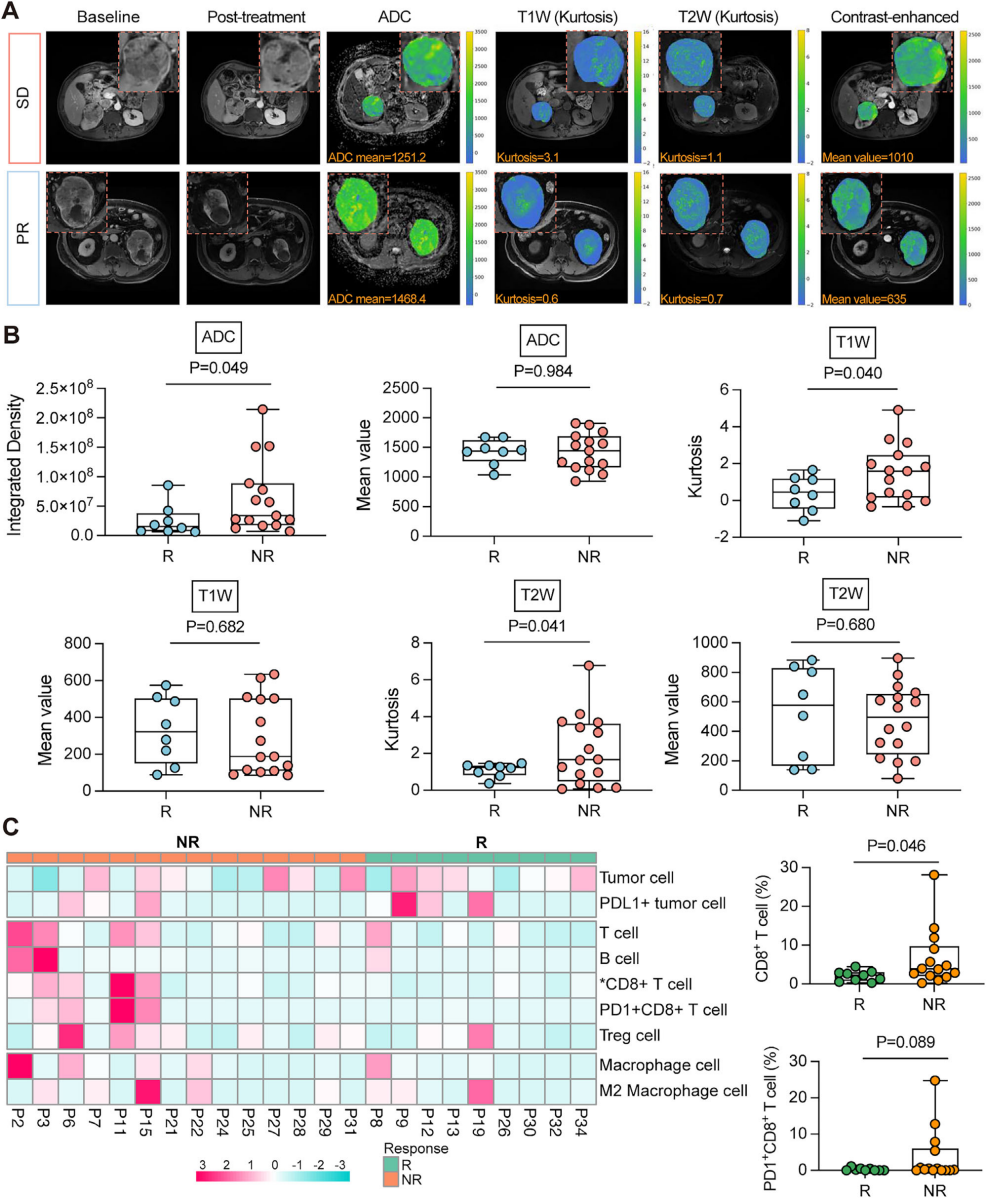
Association of baseline radiological features and immune microenvironment with treatment response. (A) Representative multiparameter MRI sequences (ADC, T1W, T2W, and arterial Phase T1) of SD and PR patients at baseline and 12 weeks. (B) Box plot of baseline multiparametric MRI measurements. The sequences and parameters displayed in the figure are as follows: ADC (integrated density and mean intensity), T1W(kurtosis and mean intensity), and T2W (kurtosis and mean intensity). Data are presented as mean ± s.e. (C) Based on immunofluorescence assays, the heatmap visualizes differential immune cell infiltration between responders and nonresponders (an intuitive comparison of infiltration patterns across individual patients (rows) and cell subgroup (columns)), while the bar chart separately depicts group‐wise differences in proportions of CD8^+^ T cells and PD‐L1^+^CD8^+^ T cells. *p < 0.05. MRI, magnetic resonance imaging; ADC, apparent diffusion coefficient; T1W, T1‐weighted; T2W, T2‐weighted; PR, partial response; SD, stable disease; R, responder; NR, nonresponder.

The multiparametric magnetic resonance imaging (MRI) images revealed distinct feature profiles according to therapeutic efficacy. Notably, responders displayed significantly elevated values of T1W/T2W kurtosis (*p* = 0.040; *p* = 0.041) and integrated density of apparent diffusion coefficient (ADC) (*p* = 0.049) compared with nonresponders (Figure 3A,B). However, the mean ADC value and dynamic contrast‐enhanced (DCE)–MRI parameters at baseline did not differ significantly between the two groups (Figures 3B and S2). In this exploratory analysis, multiparametric MRI demonstrated the potential for the early detection of tumor response to IBC in ccRCC–VTT.

**FIGURE 4:**
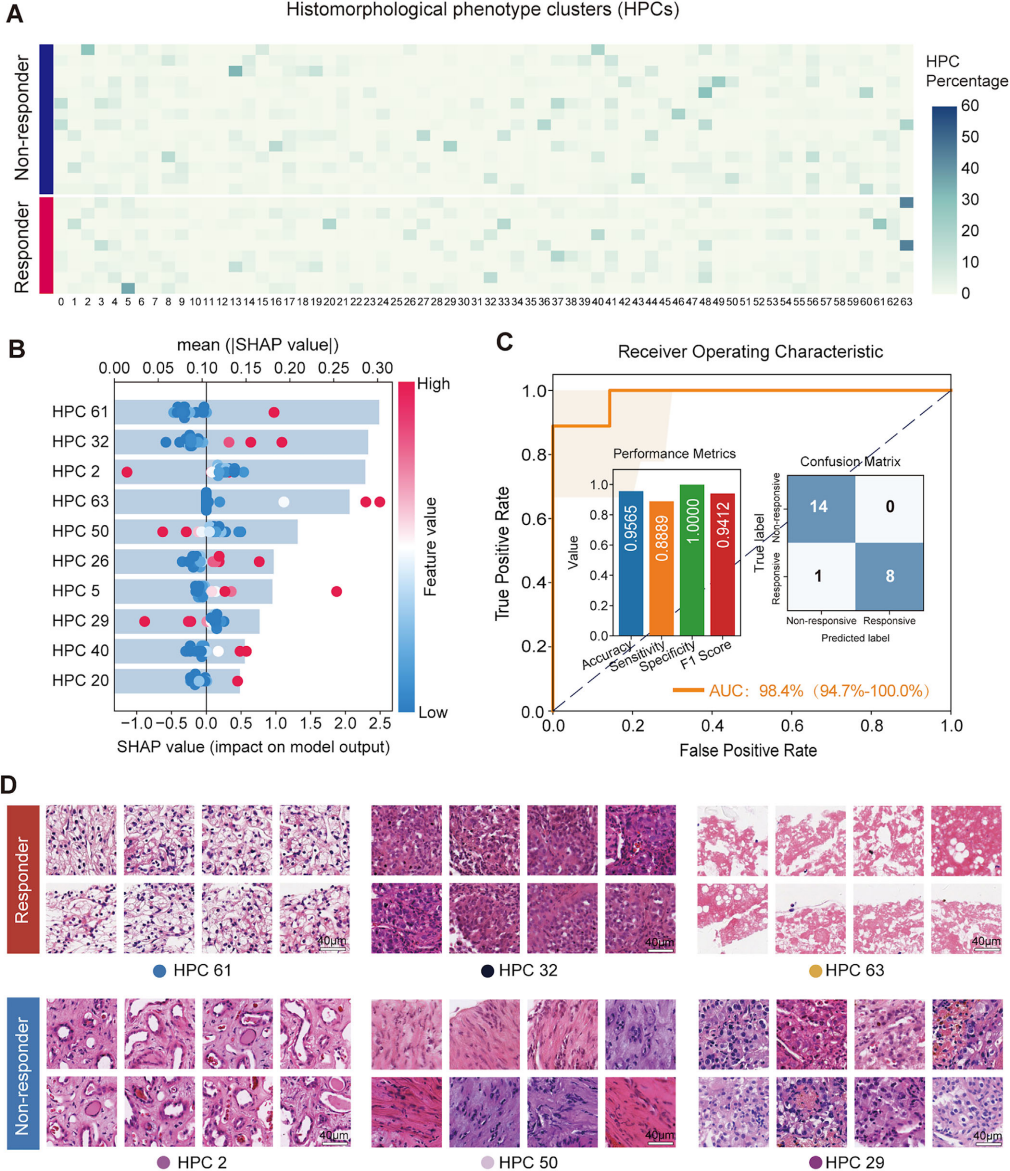
Association of baseline pathological features with treatment response. (A) The proportional patterns of histopathological phenotype clusters (HPCs) in each patient. (B) The 10 most important HPCs associated to treatment response were summarized using the interpretable SHAP to quantify the contribution value to the model's prediction. Each dot represents an individual patient. Feature values are color‐coded (red: high, blue: low). The position along the *x*‐axis indicates the feature's influence on treatment response. Points on the right push the prediction toward positive treatment response, while points on the left push toward nonresponse. The central line denotes a neutral contribution. (C) Prediction model evaluation. Receiver operating characteristic curve (ROC), performance metrics (accuracy/sensitivity/specificity/F1), and confusion matrix predicting “response” versus “nonresponse” outcomes. (D) Visualization of the six most important HPCs associated to treatment response (scale bars: 40 µm; 20×). Description of histological features of each HPC are provided in Table S2. HPCs, histopathological phenotype clusters; SHAP, Shapley Additive Explanations.

According to the pathomics analysis, 64 histomorphological phenotype clusters (HPCs) were identified and visually represented in a UMAP plot (Figures 4A and S1A). The 10 most important HPCs associated with tumor response were summarized using interpretable SHapley Additive ExPlanations (SHAP) based on the deep learning method (Figure 4B). Pathological evaluation revealed that the histological features of HPCs in responders included low‐grade ccRCC and necrosis, whereas HPCs in nonresponders manifested as high‐grade ccRCC and tumor stroma with enriched extracellular matrix (ECM) components (Figure 4D). The results based on the machine learning approach showed similar histological features associated with treatment response (Figure S1E). Our preliminary exploratory findings suggested the ECM may play critical roles in the resistance to IBC therapy. The pathomics‐based predictive model, developed through two algorithmic approaches, demonstrated good predictive power for neoadjuvant therapeutic responses (Figures 4C and S1D). Immunofluorescence analysis (Figures 3C and S3) revealed a significantly higher proportion of CD8^+^ T cell infiltration in nonresponders (*p* = 0.046). Although the CD8+/PD‐1+ T cell showed a trend toward a higher proportion in the nonresponders, the difference did not reach statistical significance (*p* = 0.089).

### 2.6 Follow‐Up and Subgroup Analyses

**FIGURE 5:**
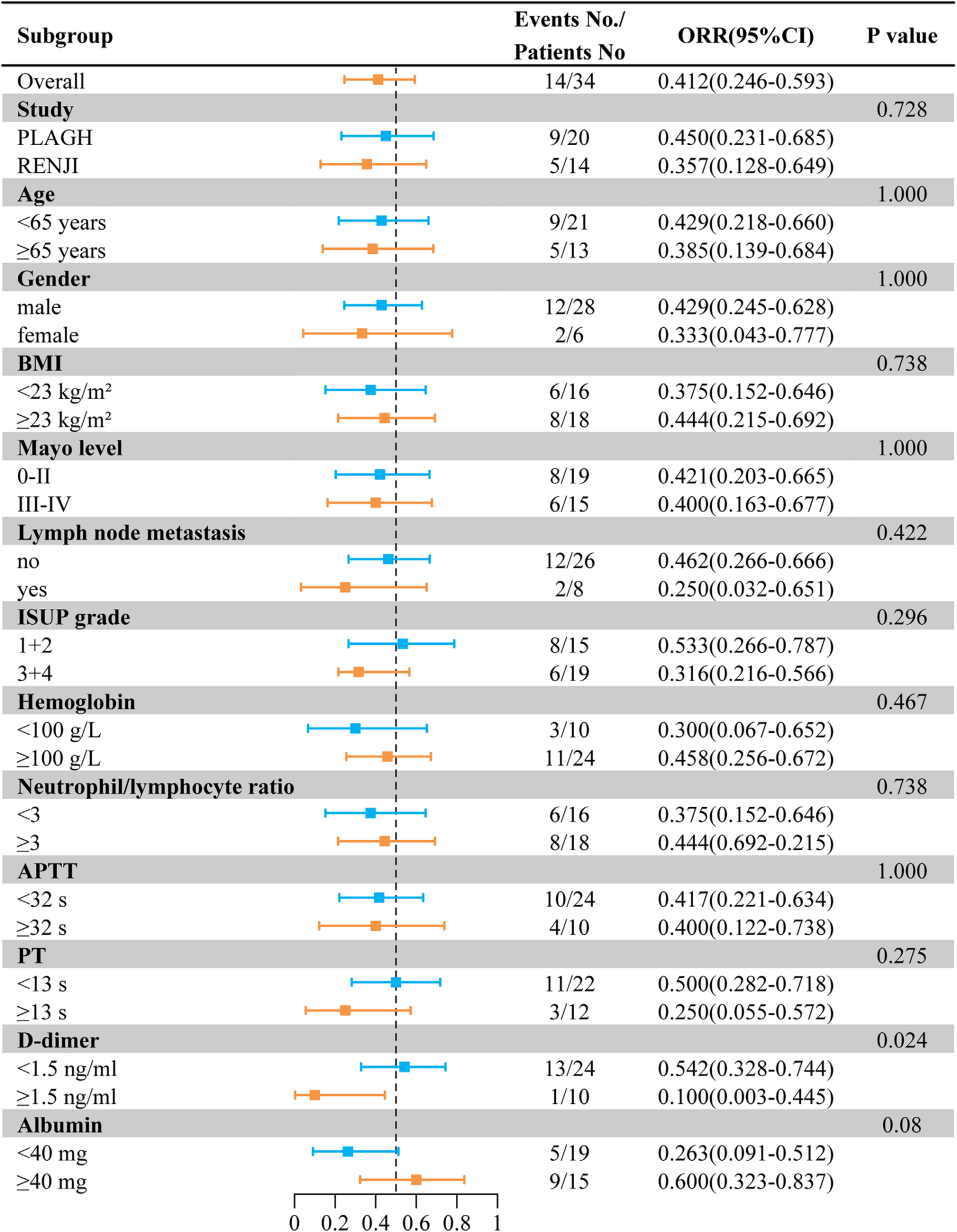
Forest plot of the ORRs in key subgroups. The subgroups were stratified by baseline patients’ characteristics. The confirmed ORR was evaluated in the key prespecified subgroups.

With a median follow‐up of 36.1 (IQR: 25.0–44.9) months, the median disease‐free survival (DFS) and overall survival (OS) were not reached (Table 2). The 1‐year DFS and OS were 76.7% (95% CI, 59.1–88.2%) and 91.2% (95% CI, 77.0–97.0%), respectively (Figure 2D,E). Five patients were dead at the time of analysis (three died of progression of ccRCC, one died of COVID‐19, and one died of Grade V postoperative complication). Subgroup analyses indicated a significantly lower ORR in patients with higher d‐dimer levels (≥1.5 ng/mL) (0.542 vs. 0.100, *p* = 0.024) (Figure 5).

## 3 Discussion

To our knowledge, this study represents the largest investigation to evaluate neoadjuvant IBC treatment in the management of patients with all levels of nonmetastatic ccRCC–VTT. Our exploratory study demonstrated promising efficacy, with a downstaging rate of 47%, an ORR of 41%, and manageable safety profile. Multiomics analysis revealed that tumor heterogeneity and a stroma‐characterized tumor microenvironment may play an important role in resistance to IBC. Subgroup analyses indicated that plasma D‐dimer levels before treatment were associated with tumor response. Currently, two Phase II clinical trials (NEOTAX and NCT04118855) have provided early evidence of neoadjuvant toripalimab plus axitinib [11, 12], although they differed in patient selection. To generate more generalizable conclusions, we performed a combined analysis from both trials in nonmetastatic ccRCC–VTT, evaluating the efficacy and safety of neoadjuvant toripalimab plus axitinib. This combined analysis not only doubled the available sample size, allowing for subgroup analyses, but also broadened the potential applicability of this neoadjuvant strategy. We further assessed baseline radiologic and pathologic features to identify multiomics determinants of treatment response. Overall, this combined analysis demonstrated that neoadjuvant toripalimab plus axitinib showed a high efficacy in downstaging the thrombus level, simplifying surgical complexity, and mitigating perioperative risks, which provides potential evidence for expanding the indication population of neoadjuvant therapy in ccRCC–VTT.

Given the high perioperative risk and technical complexity associated with VTT, researchers have prioritized preoperative systemic therapies to reduce thrombus length and increase surgical safety. Despite continuous research efforts exploring the feasibility of neoadjuvant TKI monotherapy since 2007, available evidence and consequent recommendations are still scarce in this field. A meta‐analysis had revealed limited thrombus downstaging rates (<30%) with neoadjuvant TKIs [7]. The latest Phase II study (NAXIVA trial) on neoadjuvant TKI monotherapy evaluated the efficacy of axitinib in patients with VTT. At last, 35% (seven out of 20) patients had a reduction in Mayo level and 41% (seven out of 17) patients underwent less invasive surgery than originally planned [13].

The immunotherapy revolution has redefined the RCC treatment paradigms. Based on the advancements in advanced RCC, researchers have hypothesized that combining TKIs with ICIs may enhance the efficacy of preoperative therapy. Several trials evaluating neoadjuvant TKIs/ICIs combinations have reported varying response rates. The NEOAVAX trial demonstrated a 30% (12 out of 40) ORR in localized RCC using 12 weeks of neoadjuvant avelumab plus axitinib [14]. However, another Phase II trial evaluating 8 weeks of neoadjuvant sitravatinib plus nivolumab in 20 patients with locally advanced ccRCC showed an ORR of 11.8% [15]. In this study, our combined analysis demonstrated promising efficacy of ORR, indicating that neoadjuvant toripalimab plus axitinib is a safe and effective option for patients with nonmetastatic ccRCC–VTT. The ORR in our cohort was lower than that reported in Phase III advanced RCC trials (ORR up to 56.7%) [16]. This difference is primarily attributable to the shorter duration of neoadjuvant therapy and the relatively lower neoantigen exposure burden in our cohort compared with metastatic disease. Additionally, less pronounced regression in the longitudinal dimension of the tumor thrombus, compared with transverse reduction may also have contributed to the difficulty in achieving a tumor response.

A primary concern regarding neoadjuvant therapy is disease progression during treatment. Although the disease progression rate was approximately 5.9–36% after preoperative sunitinib therapy in patients with metastatic RCC [17], progressive disease (PD) during neoadjuvant therapy was rarely reported in localized RCC [18]. Given the high tumor burden and metastatic propensity in patients with VTT, they are at high risk of PD during neoadjuvant therapy. The meta‐analysis indicated an approximately 9.9% PD rate in VTT cohorts receiving neoadjuvant therapy [7]. In our study, one (3%) patient experienced PD with an upgrade in the thrombus level. In the NAXIVA trial, reported two (11%) patients had PD during treatment [13]. Thus, vigilant monitoring for VTT during neoadjuvant therapy remains imperative. We designed an interim assessment for patients at the sixth week of treatment to identify potential drug‐resistant cases early and actively perform surgical intervention. Meanwhile, axitinib has a relatively short half‐life, which reduces the waiting time from preoperative drug discontinuation until surgery.

Despite meaningful treatment activity with neoadjuvant IBC therapy, 59% patients failed to achieve clinical benefit (defined as nonresponders) in our study. How to predict therapeutic response for personalized treatment is an important objective in oncological research. We preliminarily explored baseline radiological/histopathological features of patients with different therapeutic responses to IBC using radiomics, pathomics, and immunofluorescence analyses. Radiomic profiling revealed significant intratumoral heterogeneity with significantly elevated values of T1W/T2W kurtosis in nonresponders, which is supported by previous studies [19, 20]. Pathomic evaluation further identified aggressive histopathological phenotypes in the no‐response cohort, characterized by a high tumor malignancy grade and ECM deposition. This observation aligns with the established mechanistic evidence that densely remodeled ECM creates physical barriers to impede T‐cell trafficking and drug penetration, directly contributing to acquired resistance in both targeted and immune‐based therapies [21]. Additionally, the interaction between tumor cells and the immune microenvironment plays a central role in drug resistance. As the nuclear grade of the tumor increases, CD8+ T‐cell infiltration also increases, accompanied by a markedly exhausted phenotype, indicating the crucial role of the immunosuppressive microenvironment in malignant invasion and immune resistance in renal tumors [22, 23]. Meanwhile, in hepatocellular carcinoma patients with a pretreatment tumor necrosis rate ≥50%, neoadjuvant treatment with the PD‐1 inhibitor cemiplimab resulted in a significant pathological necrosis rate (>70%) of 45% and a 2‐year postoperative recurrence rate of only 18%. These outcomes suggest that higher baseline intratumoral immune cell infiltration (e.g., CD8^+^ T cells, dendritic cells) in tumors with more pretreatment necrosis is associated with more durable responses to immunotherapy [24]. Immunofluorescence analysis revealed a significant increase in the infiltration of exhausted CD8+PD‐L1+ cytotoxic T cells in the resistant group, which indirectly corroborates the pathological findings. Notably, the biomarker exploration in the NAXIVA study corroborates the centrality of the immune microenvironment as a potential predictor of neoadjuvant axitinib response by identifying plasma cytokines involved in T‐cell proliferation and activation (CCL17 and IL‐12) [25]. The findings from the multiplex immunofluorescence exploration are consistent with those of the present study.

Some laboratory parameters have been extensively validated for prognostic stratification in RCC, exemplified by the International Metastatic Renal Cell Carcinoma Database Consortium criteria, while their utility as predictive biomarkers for treatment response remains inadequately characterized. Elevated D‐dimer levels have been observed in thrombosis, inflammation, and tumor progression. Recent studies have suggested a potential link between increased d‐dimer levels and resistance to immunotherapy [26], while the exact mechanisms remain unclear. One plausible explanation is that elevated D‐dimer levels may reflect a heightened state of systemic inflammation or coagulopathy, which could contribute to an immunosuppressive tumor microenvironment, for example D‐dimer promotes an immunosuppressive microenvironment by activating the IL‐8/CXCL8 and MCP‐1/CCL2 pathways, which stimulates the secretion of chemokines such as CCL2 and CXCL2 to recruit myeloid‐derived suppressor cells, thereby impairing the efficacy of tumor immunotherapy [27, 28]. In all, our multiomics and subgroup analyses identified some baseline signatures with neoadjuvant IBC efficacy. Given the exploratory nature and high dimensionality with low sample size feature of multiomics analysis, our results are highly susceptible to overfitting and should be interpreted as hypothesis‐generating. Further investigation is required to determine whether these signatures can be used as predictive biomarkers of IBC resistance and to explore the underlying biological mechanisms.

Another major risk of neoadjuvant therapy is the occurrence of Grade 3–4 TRAEs that may lead to delayed surgery or treatment termination [29]. The incidence of Grade ≥ 3 TRAEs ranges from 30 to 80% after neoadjuvant TKI monotherapy, primarily manifesting as hypertension and hand–foot syndrome. Most adverse events can be alleviated by dose adjustment or drug discontinuation [30, 31]. Recently, safety data related to neoadjuvant immunotherapy have garnered increasing attention. In studies of ICIs as single‐agent neoadjuvant therapy, Gorin et al. [32] reported that two patients (11.7%) experienced Grade 3 adverse events and all patients ultimately completed three cycles of nivolumab therapy without delayed surgery. Another study by Carlo et al. [33] showed that two patients (11.1%) prematurely terminated immunotherapy because of immune‐related adverse events (irAEs) requiring hormone therapy, but none of the cases resulted in delayed surgery. However, a dual ICIs combination therapy was prematurely terminated due to a high incidence of irAEs [34]. Contrary to widely contentious safety about dual ICIs combination, published data show that the safety of neoadjuvant TKIs/ICIs combination is manageable. In the NEOAVAX trial, the incidence of Grade 3 TRAEs was 15% (six out of 40) [14]. Only one patient experienced hypothyroidism, leading to a 3‐week delay in surgery. In this exploratory analysis, the combination regimen showed an acceptable safety profile and adherence. Grade 3 adverse events were reported in 24% (eight out of 34) patients, and only one patient experienced surgical delay due to hyperglycemia, indicating that neoadjuvant toripalimab plus axitinib is a safe regimen.

Some limitations should be acknowledged. First, the two clinical trials differed in the number of toripalimab treatment cycles (three cycles at Renji Hospital vs. four cycles at PLAGH cohort). Although a significant difference existed in the number of immunotherapy cycles between the two center cohorts, subgroup analysis revealed no significant association of either the treatment cycles or the treatment center with therapeutic efficacy (Figures 5 and S4). Previously published single‐arm clinical studies have shown that the two cohorts had comparable ORRs (44 vs. 45%). Furthermore, multivariable analysis showed that neither the treatment center nor the number of therapy cycles was a predictor of treatment efficacy (Table S6). These results support the validity of pooling data from both centers. Second, surgical approaches differed across institutions. Considering the primary endpoint focused on preoperative radiological objective response and standardized surgical decision based on Mayo Clinic classification in the two cohorts, this is not so relevant for the overall evaluation of the present study. Finally, the combined analysis remains inherently limited by its single‐arm, nonrandomized design. Although the combined analysis increased the overall sample size, the relatively limited cohort and inherent heterogeneity between the two studies may introduce unmeasured confounders. Our findings regarding efficacy, subgroup analyses and multiomics findings were preliminary exploratory results in this high‐risk group. The limited median follow‐up constrains a definitive assessment of long‐term survival outcomes. Further randomized controlled studies are necessary to confirm the efficacy and safety of neoadjuvant IBC therapy in patients with ccRCC–VTT.

## 4 Conclusion

Neoadjuvant toripalimab plus axitinib showed promising clinical efficacy and safety in downstaging thrombus level and simplifying surgical complexity for VTT, providing a new strategy for the perioperative management of ccRCC with VTT. Multiparametric MRI, histopathological examination, and plasma D‐dimer levels prior to treatment demonstrated the potential for early detection of tumor response to neoadjuvant IBC. Randomized clinical trials with long‐term follow‐up are needed to further validate these findings and evaluate the long‐term benefits of neoadjuvant IBC therapy.

## 5 Materials and Methods

### 5.1 Study Design and Participants

These two Phase II, open‐label, single‐arm trials (NEOTAX and NCT04118855) were designed to assess the efficacy and safety of neoadjuvant toripalimab plus axitinib in patients with locally advanced ccRCC. Written informed consent was obtained from all participants before enrollment. The study protocol was approved by the institutional review boards of the PLAGH and Renji Hospital. The two trials were registered separately in the Chinese Clinical Trial Registry (ChiCTR2000030405) and clinicaltrials.gov (NCT04118855).

The target population of this study comprised patients with RCC–VTT. The key eligibility criteria included: (1) age ≥18 years; (2) Mayo Level 0–IV tumor thrombus (cT3a/b/c‐T4 stage) without metastatic disease (M0); (3) candidates for radical nephrectomy and tumor thrombectomy with a life expectancy of ≥3 months; (4) histopathologically confirmed ccRCC; (5) Eastern Cooperative Oncology Group performance status 0–1; and, (6) adequate hematologic and organ function (detailed diagnostic criteria refer to the protocol). The key exclusion criteria were as follows: (1) prior systemic anticancer therapy (including TKIs or ICIs); (2) presence of any other cancers within the past 5 years; and (3) presence of surgical contraindications.

### 5.2 Procedures

The eligible patients received toripalimab in combination with axitinib. The dose of axitinib was 5 mg BID. Toripalimab was administered via 60‐min intravenous infusions at 240 mg every 3 weeks (q3w). According to the trial protocols, both trials allowed for dose reduction of axitinib. However, the two clinical trials differed in the number of toripalimab treatment cycles: the NEOTAX trial administered toripalimab as a neoadjuvant therapy for four cycles, whereas the NCT04118855 trial maintained a maximum of three cycles. The patients underwent scheduled imaging reassessment at Weeks 6 and 12 of treatment and preoperative evaluation of the radiological response.

### 5.3 Endpoints

The primary endpoint was radiographic objective response prior to surgery assessed using RECIST version 1.1 criteria. The ORR was determined by a comprehensive assessment of tumor response based on the maximum diameter of the PT and the length of the VTT. Secondary endpoints included the downstaging rate of VTT based on the Mayo level, change in thrombus length, postoperative complication (Clavien‐Dindo classification), change in surgical plan, DFS, OS, and safety. For patients with baseline Mayo Level I–IV tumor thrombus, the downstaging of tumor thrombus was defined as a reduction in Mayo Clinic classification after 12 weeks of treatment. For patients with Mayo Level 0 thrombus, the definition of downstaging differed depending on the laterality of PT. In right RCC with VTT, downstaging was defined when the proximal end of the VTT retracted from the main renal vein to the branches of the renal vein. In left‐sided cases, the superior mesenteric artery (SMA) was used as an anatomical landmark. Downstaging was defined when the VTT retracts from the lateral to the abdominal aorta to the level of the SMA or from the main renal vein lateral to the SMA to the branches of the renal vein.

### 5.4 Surgery and Follow up

The surgical strategy was determined primarily based on the thrombus level, as previously described [35]. The choice of surgical approach was determined by surgeons' experience, patients' surgical history, and available equipment platform. The surgical approach modification rate was quantified by analyzing the discrepancies between the preoperatively planned and intraoperatively executed strategies, as previously described. Based on Mayo classification and specific anatomical landmarks, we divided venous tumor thrombectomy into eight different surgical strategies (strategy A–H). With the upgrading of surgical strategy classification, the surgical complexity and step count increased. The definitions of strategy classification and key points of surgical strategy are presented in Table S3.

Patients were followed up every 3 months for the first 5 years and every 6 months thereafter until postoperative death. Follow‐up chest, abdominal, and pelvic contrast‐enhanced computed tomography or MRI was performed every 3 months or upon suspected tumor progression.

### 5.5 Multiomics Analyses

*MRI analysis*: Twenty‐four patients underwent baseline multiparametric MRI prior to neoadjuvant therapy. All examinations were performed on a 3.0‐T clinical MRI system (Discovery MR750; GE Healthcare, USA) using a phased‐array body coil for signal reception. The imaging protocol comprised the following sequences: axial T2‐weighted imaging (T2WI), axial T1‐weighted imaging (T1WI), DWI with subsequent ADC map generation, and DCE–T1WI. These baseline parameters, measured before neoadjuvant therapy, reflect tumor cellularity, vascularity, and heterogeneity within individual lesions and were evaluated for their correlation with therapeutic efficacy. All images were reconstructed using the vendor‐provided reconstruction pipeline. Key acquisition parameters for each sequence are summarized in Supporting Information: methods to ensure reproducibility.

All sequences were imported into 3D Slicer (version 5.6.1). The primary renal tumor and VTT were manually delineated slice‐by‐slice on each modality separately by an experienced radiologist blinded to tumor response (experience 7 years). A kidney mask was also manually defined on each modality. No intermodality registration was performed because ROIs were independently segmented on each sequence, and features were extracted within the corresponding sequence‐specific ROIs. To reduce intersubject intensity scaling variability, *z*‐score normalization was applied within the kidney mask for each modality: I_norm = (I‐μ_“kidney”)/σ_“kidney” (the cystic and necrotic areas were manually excluded from the solid tumor ROI). For each ROI on T2WI, T1WI, ADC, and DCE–T1WI, five features were computed: voxel count, mean intensity, integrated density, kurtosis, and histogram entropy.

*Pathomics analysis*: We analyzed the diagnostic pathology on hematoxylin and eosin stained whole‐slide images (WSIs) of PT biopsy specimens from 23 patients. WSIs were generated from archival formalin‐fixed paraffin‐embedded tissue blocks and digitized at 40× magnification using the KF‐SCAN‐ST model slide scanner (Jiangfeng Biotechnology Co., Ltd.). Images were converted to the SVS format. For standardized downstream processing, WSIs were downsampled to 20× resolution and partitioned into nonoverlapping 224 × 224‐pixel tiles. Our model applies data augmentations to construct data pairs, encodes them with a shared‐weight, dual‐branch ResNet50, and applies two MLP heads to learn tile‐ and cluster‐level representations. A joint tile‐ and cluster‐level contrastive objective is used to learn representations and partition tiles into 64 HPCs. The encoder was trained from scratch without ImageNet/histology pretraining for 100 epochs (batch size 32; AdamW with cosine annealing; PyTorch v1.11.0) with no pretraining. A deep learning‐based contrastive clustering model was used to identify distinct HPCs, and the proportional patterns are shown in Figure S1. Our framework learns feature representations and cluster assignments simultaneously, rather than clustering after representation learning. During training, the network iteratively updates both the feature representations and the cluster assignments under the joint contrastive‐and‐clustering losses and converges to the predefined 64 morphological clusters. Two distinct algorithmic approaches were used to investigate the connection between these patterns and the treatment response. First, deep learning with a transformer encoder was implemented to transform the quantified pathomic features into optimized representations for treatment response prediction. The machine learning method utilized LASSO regression with 10‐fold cross‐validation to automatically select the optimal lambda parameter. We identified key features based on the lambda‐optimized model and developed a regression model.

*Immunofluorescence analysis*: To comprehensively profile the tumor immune microenvironment, we simultaneously assessed the following markers on consecutive tissue sections: Pan‐Cytokeratin (tumor cells), PD‐L1, CD3 (T cells), CD20 (B cells), CD8 (cytotoxic T cells), PD‐1, FoxP3 (regulatory T cells, Tregs), CD68 (macrophages), and CD163 (M2‐like macrophages). Multiplexed imaging was performed using the Cell DIVE whole‐slide imaging platform (Leica Biosystems). This system utilizes an iterative, multiround staining and imaging process. Briefly, in each round, a subset of markers (typically 3–4) was labeled with fluorescent dyes, imaged at 20× magnification, and then the fluorophores were chemically inactivated (“erased”). This cycle was repeated with the next antibody subset until all markers in the panel were captured. The final output is a coregistered, multichannel WSI containing the signal for all markers.

All quantitative analyses were performed using Cell DIVE Analysis Toolbox and Indica Labs’ HALO image analysis platform (specifically, the HALO AI and multiplex IHC modules). Nuclei were primarily segmented based on the DAPI signal using a deep learning‐based nuclear detection algorithm within HALO AI. The algorithm was trained on a representative subset of our images to ensure accurate detection across varying tissue and staining qualities. Cytoplasm and membrane boundaries for each cell were defined by expanding from the detected nuclei, utilizing the signal from relevant markers (e.g., cytokeratin for tumor cells). Following segmentation, cells were classified into specific phenotypes based on the coexpression patterns of the markers. Boolean logic gates were applied to the fluorescence intensity data for each marker channel. For example: CD8+ T cell: CD3+ AND CD8+; PD‐1+CD8+ T cell: CD3+ AND CD8+ AND PD‐1+; Treg: CD3+ AND FoxP3+; M2 macrophage: CD68+ AND CD163+. The primary quantitative outputs were: (1) the density (cells per mm^2^) of each phenotypically defined cell population and (2) the percentage of specific subsets (e.g., %PD‐1+ cells among all CD8+ T cells). These metrics were calculated for the entire tumor region, which was manually annotated by a pathologist on each WSI.

Patients achieving partial response (PR) were categorized as responders, while those with PD or SD were considered nonresponders.

### 5.6 Statistical Analysis

Differences between continuous variables were tested using the *t*‐test or Mann–Whitney *U* test. The chi‐square test or Fisher's exact test was used to compare categorical data. Survival was estimated using the Kaplan–Meier method and assessed the difference with log‐rank test. All statistical analyses were performed using SPSS software (version 26.0), with *p* < 0.05 considered statistically significant.

## Author Contributions

Cheng Peng, Liangyou Gu, and Jiwei Huang had full access to all of the data in the study and take responsibility for the integrity of the data and the accuracy of the data analysis. Conception and design: L. Gu, J. Huang, X. Ma, X. Zhang, and C. Peng. Acquisition of data: C. Peng, Z. Wu, Y. Wang, Q. Liang, D. Wang, and H. Zhao. Analysis and interpretation of the data: C. Peng, Y. Wang, J. Li, X. Yue, J. Song, C. Shi, and Y. Zhang. Drafting of the manuscript: C. Peng, Y. Wang, Y. Zhang, and L. Gu. Critical revision of the manuscript for important intellectual content: L. Gu, B. Wang, G. Yang, Q. Huang, H. Wang, X. Ma, and X. Zhang. All authors have read and approved the final manuscript.

## Funding

The project was supported by the National Natural Science Foundation of China (No. 82373169，82573476 and 82273412), the Beijing Natural Science Foundation (No.L248017) and Chinese PLA General Hospital Youth Program (No. 22QNCZ021).

## Ethics Statement

This study was approved by the Ethics Committee of the Chinese PLA General Hospital and RENJI Hospital (Internal Registration No. S2019‐349 and No. KY2019‐131). All patients provided written informed consent.

## Conflicts of Interest

The authors declare no conflicts of interest.

## Clinical Trial Registration

The two trials were registered separately in the Chinese Clinical Trial Registry (No. ChiCTR2000030405) and clinicaltrials.gov (NCT04118855).

## Supporting information



Supplementary information

## Data Availability

All data used to support the findings of this study are available in the manuscript and its Supporting Information files. The corresponding authors will examine all requests for additional data sharing to determine whether intellectual property or confidentiality obligations apply.
