# Supplementary material for: Neoadjuvant Toripalimab Plus Axitinib for Nonmetastatic Clear Cell Renal Cell Carcinoma With Tumor Thrombus: A Combined Analysis of Two Phase II Clinical Trials
Source: MedComm (2020). 2026 Apr 6;7(4):e70720. doi: 10.1002/mco2.70720 (PMC13053665; doi:10.1002/mco2.70720)
Supplement: Supplementary file 1 — Supplementary information [file MCO2-7-e70720-s001.docx]

**Supplemental Material**

**Title: Neoadjuvant toripalimab plus axitinib for nonmetastatic clear cell renal cell carcinoma with tumor thrombus: a combined analysis of two phase II clinical trials**

**Authors:**

Cheng Peng^1^^#^, MD; Zhuolong Wu^2#^, MS; Yaohui Wang^1#^, MD; Qiyang Liang^1,6^, MS; Dongxing Wang^1^, MS; Houming Zhao^1,6^, MS; Jinhang Li^7^, MD; Xiuzheng Yue^4^, MS; Yibo Zhang^5^, MS; Jialong Song^1,8^, MS; Changwei Shi^1,8^, MS; Haiyi Wang^3^, MD; Guoqiang Yang^1^, MD; Baojun Wang^1^, MD; Qingbo Huang^1^, MD; Xu Zhang^1*^, MD; Xin Ma^1*^, MD; Jiwei Huang^2*^, MD; Liangyou Gu^1*^,MD

**Affiliations：**

1 Senior Department of Urology, Chinese PLA General Hospital, Beijing, China

2 Department of Urology, RenJi Hospital, Shanghai Jiao Tong University, School of Medicine, Shanghai, China

3 Department of Radiology, The First Medical Center, Chinese PLA General Hospital, Beijing, China

4 Medical Big Data Research Center, Medical Innovation Research Division of PLA General Hospital, Beijing, China.

5 School of Biomedical Engineering, Wenzhou Medical University, Wenzhou, China.

6 Chinese PLA Medical School, Chinese PLA General Hospital, Beijing, China.

7 Department of Pathology, The First Medical Center, Chinese PLA General Hospital, Beijing, China

8 School of Medicine, Nankai University, Tianjin, China

**Corresponding authors:**

Liangyou Gu

Senior Department of Urology, Chinese PLA General Hospital, No. 69, Yongding Road, Haidian District, Beijing, China

Post Code: 100039

Email: guliangyouyd1@126.com

Jiwei Huang

Department of Urology, RenJi Hospital, Shanghai Jiao Tong University, School of Medicine, No. 160, Pujian Road, Pudong New District, Shanghai, China

Post Code: 200127

Email: huangjiwei@renji.com

Xin Ma

Senior Department of Urology, Chinese PLA General Hospital, No. 69, Yongding Road, Haidian District, Beijing, China

Post Code: 100039

Email: mxin301@126.com

Xu Zhang

Senior Department of Urology, Chinese PLA General Hospital, No. 69, Yongding Road, Haidian District, Beijing, China

Post Code: 100039

Email: xzhang@tjh.tjmu.edu.cn

**Supplemental Methods**

Key acquisition parameters for each MRI sequence

Pathomics analysis of H&E-stained whole-slide images

**Supplementary tables and figures**

Table S1. TRAEs in all patients

Table S2. Description of histological features of each HPC

Table S3. The detailed evaluation of assessing surgical complexity

Table S4. A comparison of the profiles of three neoadjuvant studies in renal cell carcinoma

Table S5. Description and the difference comparison of the statistical designs and inclusion/exclusion criteria between NEOTAX trial and NCT04118855 trial

Table S6. Multivariable analysis of objective response rate

Figure S1. Discovery and visualization of histomorphological features associated with treatment response based on machine learning method

Figure S2. Box plot of additional baseline multiparametric MRI measurements between responders and non-responders

Figure S3. Representative images of the immunofluorescence analysis.

Figure S4. Comparison of immunotherapy cycles across different centers and their relationship with tumor response.

Key acquisition parameters for each MRI sequence

T2WI: TR/TE =XX ms /XX ms; flip angle = XX°; FOV = XX mm; matrix = XX; slice thickness/gap = XX mm / XX mm; acquisition plane = axial; fat suppression = XX.

T1WI: TR/TE = XX ms /XX ms; flip angle =XX°; FOV = XX mm; matrix =XX; slice thickness/gap = XX mm / XX mm; fat suppression = XX.

DWI/ADC: b-values = 0 and 800 s/mm²; TR/TE = XX ms / XX ms; slice thickness/gap = XX mm / XX mm; EPI factor = XX; parallel imaging acceleration; ADC maps were generated on the scanner console using a mono-exponential model: S(b)=S_0 exp⁡(-b⋅ADC).

DCE T1WI: temporal resolution = XX s/phase; number of phases = XX; TR/TE = XX ms / XX ms; flip angle = XX°; contrast agent = XX; dose = e.g., 0.1 mmol/kg; injection rate = XXmL/s.

Pathomics analysis of H&E-stained whole-slide images

H&E-stained whole-slide images (WSIs) were generated from archival formalin-fixed paraffin-embedded (FFPE) tissue blocks and digitized at 40× magnification. WSIs were downsampled to 20× resolution and partitioned into non-overlapping 224 × 224-pixel tiles. To remove background, we used Otsu thresholding to estimate tissue coverage per tile and retained only tiles with ≥60% tissue. This served as an initial QC step to improve signal-to-noise for downstream representation learning and phenotyping.

We trained a contrastive clustering–based self-supervised model to learn morphology-aware tile representations. Briefly, for each tile image x, we generated a positive pair by applying two independent stochastic augmentations t_1 (⋅) and t_2 (⋅), producing x ̃_1=t_1 (x) and x ̃_2=t_2 (x). The two views were encoded by a shared-weight dual-branch ResNet50 encoder f(⋅) to obtain embeddings h_1=f(x ̃_1) and h_2=f(x ̃_2). To capture morphology at different semantic levels, we further used two nonlinear MLP heads: a tile-level head g_tile (⋅) producing z=g_tile (h), and a cluster-level head g_cluster (⋅) producing cluster-oriented representations. The model was optimized using a joint objective that combines (i) a tile-level contrastive loss to maximize agreement between embeddings of the same tile under different augmentations while separating different tiles within a batch, and (ii) a cluster-level contrastive loss to encourage consistent cluster assignments and well-separated cluster structure. Training was conducted for 100 epochs (batch size 32) using AdamW with weight decay and a cosine annealing learning-rate schedule, implemented in PyTorch v1.11.0, on a single NVIDIA GeForce RTX 4080 GPU (training time ~61 hours).

Table S1. TRAEs in all patients

| TRAE | Grade 1, No. (%) | Grade 2, No. (%) | Grade 3, No. (%) | Grade 4-5, No. (%) | Total (n=34), n(%) |
| --- | --- | --- | --- | --- | --- |
| Any TRAE | 28 (82) | 24 (71) | 8 (24) | 0 | 34 (100) |
| Hypertension | 1 (3) | 8 (24) | 2 (6) | 0 | 11 (32) |
| Proteinuria | 2 (6) | 6 (18) | 2 (6) | 0 | 10 (29) |
| Fatigue | 9 (26) | 1 (3) | 0 | 0 | 10 (29) |
| Diarrhea | 2 (6) | 4 (12) | 0 | 0 | 6 (18) |
| Rash | 2 (6) | 4 (12) | 0 | 0 | 6 (18) |
| Weight decreased | 5 (15) | 1 (3) | 0 | 0 | 6 (18) |
| Dysphonia | 6 (18) | 0 | 0 | 0 | 6 (18) |
| Hypertriglyceridemia | 3 (9) | 1 (3) | 1 (3) | 0 | 5 (15) |
| PPE syndrome | 2 (6) | 1 (3) | 1 (3) | 0 | 4 (12) |
| Decreased appetite | 4 (12) | 0 | 0 | 0 | 4 (12) |
| LDH increased | 2 (6) | 2 (6) | 0 | 0 | 4 (12) |
| Voice alteration | 4 (12) | 0 | 0 | 0 | 4 (12) |
| Hypothyroidism | 0 | 3 (9) | 1 (3) | 0 | 4 (12) |
| TSH increased | 0 | 3 (9) | 0 | 0 | 3 (9) |
| ALT increased | 0 | 2 (6) | 1 (3) | 0 | 3 (9) |
| AST increased | 0 | 2 (6) | 1 (3) | 0 | 3 (9) |
| Mucositis | 0 | 3 (9) | 0 | 0 | 3 (9) |
| Blood creatine increased | 2 (6) | 1 (3) | 0 | 0 | 3 (9) |
| Stomatitis | 2 (6) | 1 (3) | 0 | 0 | 3 (9) |
| Pruritus | 3 (9) | 0 | 0 | 0 | 3 (9) |
| Constipation | 2 (6) | 0 | 0 | 0 | 2 (6) |
| Nausea | 1 (3) | 1 (3) | 0 | 0 | 2 (6) |
| Hypercholesterolemia | 1 (3) | 1 (3) | 0 | 0 | 2 (6) |
| Gamma-GT increased | 1 (3) | 0 | 0 | 0 | 1 (3) |
| Urinary tract infection | 1 (3) | 0 | 0 | 0 | 1 (3) |
| Hyperglycaemia | 0 | 0 | 1 (3) | 0 | 1 (3) |

Abbreviation: TRAE, treatment-related adverse event; AST, aspartate aminotransferase; ALT, alanine aminotransferase; PPE, palmarplantar erythrodysesthesia; LDH, lactate dehydrogenase; TSH, thyroid-stimulating hormone; GT, glutamytransferas

Table S2. Description of histological features of each HPC

| HPC | Lable | General description |
| --- | --- | --- |
| 0 | Stromal components | Proliferative fibrous tissue with scattered thin-walled capillaries |
| 2 | Stromal components | Thin-walled capillaries |
| 5 | Renal tumor | Tumor cells (WHO/ISUP 2 grade) with focal areas exhibiting hemorrhage and/or hemosiderin deposition |
| 6 | Stromal components | Proliferative stromal tissue |
| 15 | Stromal components | Hyalinized fibrous tissue containing thick-walled vessels |
| 17 | Renal tumor | Predominant tumor cells with abundant eosinophilic cytoplasm. Nuclear grade: Predominantly WHO/ISUP 2 grade with focal grade 3. |
| 21 | Necrosis | The necrotic areas often appear as pale or pink regions devoid of cellular details, with disrupted cell membranes and nuclear fragmentation. |
| 25 | Renal tumor | Tumor cells displaying intercellular spaces with WHO/ISUP nuclear grade 3 |
| 26 | Renal tumor | Tumor cells arranged in glandular patterns with abundant eosinophilic cytoplasm, WHO/ISUP nuclear grade 2 |
| 28 | Stromal components and necrosis | Dense, pink or eosinophilic material and vessels tissues. The necrotic areas were also observed. |
| 29 | Renal tumor | Tumor cells exhibiting eosinophilic cytoplasm with readily identifiable mitotic figures, WHO/ISUP nuclear grade 3 |
| 32 | Renal tumor | Tumor cells show abundant clear cytoplasm. Nuclear Grading (WHO/ISUP System) was grade 2. |
| 48 | Renal tumor | Tumor cells exhibiting eosinophilic cytoplasm with readily identifiable mitotic figures, WHO/ISUP nuclear grade 3 |
| 50 | Stromal components | Proliferative fibrous tissue |
| 53 | Renal tumor | Tumor cells with clear cytoplasm and WHO/ISUP nuclear grade 2 |
| 56 | Stromal components and tumor | Predominantly fibrous tissue with scattered tumor cells |
| 57 | Renal tumor | Tumor cells displaying intercellular spaces with WHO/ISUP nuclear grade 3 |
| 61 | Stromal components | The stromal area exhibits abundant infiltration of immune cells and tissue foam cells, focal deposits of golden-brown hemosiderin pigment |
| 63 | Necrosis | Necrotic tumor tissue |

Table S3 The detailed evaluation of assessing surgical complexity

| Surgical strategy classification | Indications | Definition and key points of surgical strategy |
| --- | --- | --- |
| Strategy H | Level IV VTT: thrombus extending into right atrium | Establishment of cardiopulmonary bypass;  Thoracoscopy-assisted atriotomy for thrombectomy |
| Strategy G | Level IV VTT: thrombus extending above the diaphragm, but not into right atrium | Without cardiopulmonary bypass;  Intrapericardial IVC clamp;  Excision of infradiaphragmatic thrombus similar as Level III |
| Strategy F | Level III VTT: between the second porta hepatis (SPH) and diaphragm | Both mobilization of right and left lobes of the liver;  Clamping of suprahepatic and infradiaphragmatic IVC and FPH;  Other surgical procedures similar as Level II with strategy E |
| Strategy E | Level II VTT: between the first porta hepatis (FPH) and SPH | Mobilization of right lobe of the liver under liver mobilization position;  Additional SHVs (3-5) ligation;  Reposition for thrombectomy;  Other surgical procedures similar as Level II with strategy D |
| Strategy D | Level II VTT: thrombus into IVC ≥2 cm and below SPH. | Mobilization of right lobe of the liver under thrombectomy position;  SHVs ligation (typically 1-3) ;  Other surgical procedures similar as strategy C |
| Strategy C | Level I VTT: thrombus into IVC < 2 cm  from renal vein ostium level | Retraction of the liver;  Exposure of IVC;  Incision of IVC and IVC repair |
| Strategy B | Level 0 VTT: the VTT invades into the main renal vein for right side cases or beyond the SMA for left cases | mobilize both the renal vein and the pararenal IVC; ligation of the renal vein and the IVC wall does not require incision |
| Strategy A | Level 0 VTT: the VTT is restricted to the branches of the renal vein or proximal of SMA | Only mobilize the renal hilum |

Abbreviation: IVC, inferior vena cava; VTT, venous tumor thrombus; SHV, short hepatic veins; FPH, first porta hepatis; SPH, second porta hepatis; SMA, superior mesenteric artery

Table S4. A comparison of the profiles of three neoadjuvant studies in renal cell carcinoma.

| Variable | Our Study | NAXIVA | NEOAVAX |
| --- | --- | --- | --- |
| Patient characteristics | High-risk non-metastatic ccRCC with TT (cT3a-4N0-1M0) | Resectable ccRCC with VTT (cT3a-3cN0-1M0-1) | High-risk non-metastatic ccRCC (cT1b-2aG4/cT2bG3/cT3aG3-4/cT3b-4Gx N0M0, cTxN1M0) |
| Treatment regimen | Axitinib + Toripalimab for up to 12weeks | Axitinib for 8weeks | Axitinib + Avelumab for 12weeks |
| ORR | 41% (14/34) | 16.7% (3/18) | 30% (12/40) |
| TT downstaging rate | 47% (16/34) | 35.0% (7/20) | / |
| Safety outcomes | Grade 3 TRAEs：23.5% (8/34) | Not reported | Grade 3 TRAEs：15.0% (6/40) |

Abbreviation: ccRCC, Clear Cell Renal Cell Carcinoma; TT, Tumor Thrombus; VTT, Venous Tumor Thrombus; ORR, Objective Response Rate.

Table S5. Description and the difference comparison of the statistical designs and inclusion/exclusion criteria between NEOTAX trial and NCT04118855 trial

| Key Parameter | NEOTAX | NCT04118855 | Difference |
| --- | --- | --- | --- |
| Study objective | The efficacy and safety of neoadjuvant IBC | The efficacy and safety of neoadjuvant IBC | NA |
| Study design | Single-arm, open-label, single-center phase II clinical trial | Single-arm, open-label, single-center phase II clinical trial | NA |
| Drug intervention | Toripalimab plus axitinib | Toripalimab plus axitinib | NA |
| Primary end point | The down-staging rate of IVC-TT level. | ORR | As the initial phase II study to assess neoadjuvant IBC treatment in managing RCC with IVC-TT, the NEOTAX trial set the down-staging rate as primary end point, which was  is closely related to the safety of surgery. Given that NCT04118855 trial specifically enrolled patients with localized and locally advanced RCC, only a subset presenting with venous tumor thrombus, this trial protocol rationally adopted ORR as its primary endpoint. |
| Statistical analysis | Simon’s two-stage minimax design: Null hypothesis (H₀): the down-staging rate of IVC-TT level ≤10%. Alternative hypothesis (H₁): True down-staging rate > 30%.Type 1 error is set to 0.1 and power is set to 0.9. | Bayesian design: The distribution of πORR follows a β distribution with parameters (0.64–1.36), yielding a mean of 0.32 and a SD of 0.27. πORR-β (0.641.36) with an average value of 0.32. It can be inferred that the efficacy of axitinib plus toripalimab in patient treatment would be deemed satisfactory if the posterior probability of πORR exceeding 0.32 is ≥80%. | All statistical methodologies were rigorously aligned with the prespecified study design framework, respectively. |
| Sample size | 25 | 20 | On the basis of the above information |
| Key inclusion criteria | Level II–IV IVC-TT (cT3b/c or T4), cN0/1, cM0/1, candidates for radical nephrectomy, and IVC thrombectomy;  Clear cell renal cell carcinoma confirmed by biopsy;  ECOG 0-1;  Adequate organ function | Non-metastatic disease(T2-T3,N0-1,M0);  Clear cell renal cell carcinoma confirmed by biopsy;  Scheduled for either partial or radical nephrectomy;  ECOG 0-1;  Adequate organ function | The NEOTAX trial enrolled patients with or without regional lymph node involvement (N0/1) and distant metastasis (M0/1), whereas the NCT04118855 trial implemented strict eligibility criteria limited to non-metastatic (M0) disease |
| Key exclusion criteria | The presence of any other cancers within the past 5 years;  previous anticancer systemic therapy including TKI or IBC;  recent history of cardiac or vascular events;  M1 participants belonging to IMDC poor-risk group. | The presence of any other cancers within the past 5 years;  the use of other systemic antitumor treatments (including targeted therapy and immunotherapy);  the requirement for immediate nephrectomy to alleviate symptoms;  current use of immunosuppressive agents, pregnancy or breastfeeding, and a history of autoimmune disease or syndrome. | Performing cytoreductive nephrectomy for metastatic RCC is a topic of controversy, particularly for poor-risk patients. Thus, the NEOTAX trial did not enroll patients with IMDC poor-risk group. |
| Drug Dose | Axitinib was maintained at 5 mg orally twice daily (BID). Toripalimab was administered via 60-minute intravenous infusion at 240 mg every 3 weeks (q3w). | Axitinib was maintained at 5 mg orally twice daily (BID) throughout treatment duration. Toripalimab was administered via 60-minute intravenous infusion at 240 mg every 3 weeks (q3w). | NA |
| Treatment cycle | Axitinib was maintained throughout treatment duration. (12 weeks). Toripalimab was administered for 4 cycles. | Axitinib was maintained throughout treatment duration. (12 weeks).  Toripalimab was administered for a maximum of 3 doses. | The two clinical trials differed in the number of Toripalimab treatment cycles: the NEOTAX trial administered Toripalimab as neoadjuvant therapy for 4 cycles, whereas the NCT04118855 trial maintained a maximum of 3 cycles. |

Table S6. Multivariable analysis of objective response rate

| Variable | Multivariable | |
| --- | --- | --- |
|  | OR (95%CI) | P |
| Centers (PLAGH vs. RENJI) | 2.228 (0.282-18.433) | 0.439 |
| Numbers of immunotherapy cycles | 1.060 (0.264-4.248) | 0.935 |
| Tumor thrombus levels | 1.280 (0.615-2.661) | 0.509 |
| ISUP grade | 2.913 (0.665-12.748) | 0.156 |

Abbreviation: ISUP, International Society of Urological Pathology.


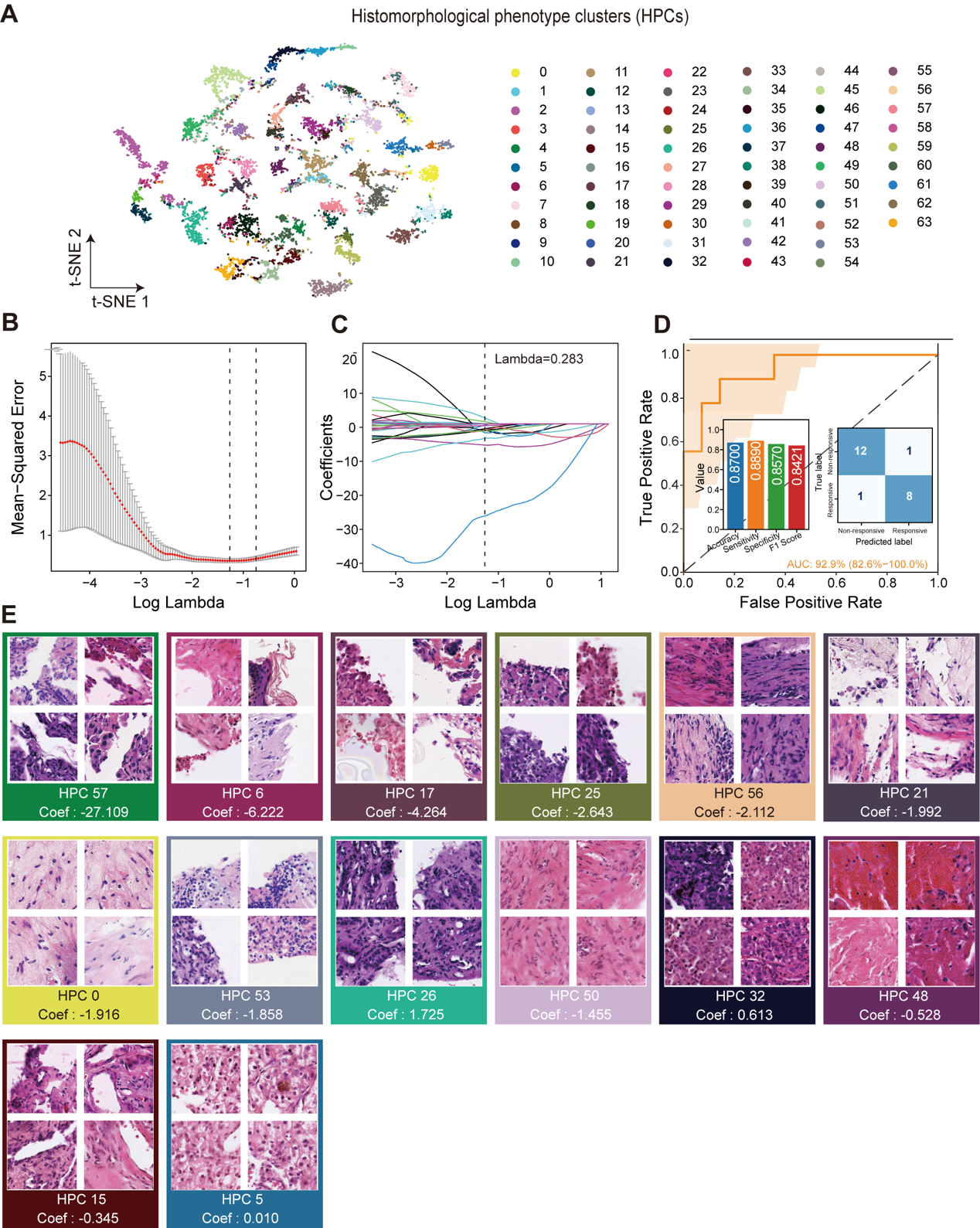


Figure S1. Discovery and visualization of histomorphological features associated with treatment response based on machine learning method

(A) t-SNE visualization of 64 distinct histopathological phenotype clusters (HPCs). (B) Cross-validated mean-squared error (MSE) versus log(λ). Red curve indicates MSE trend, gray band represents 95% confidence interval.(C) Coefficient shrinkage paths across log(λ). Each colored line denotes a feature coefficient, vertical dashed line marks optimal λ (0.185) selected by minimum MSE criterion.(D) Prediction model evaluation. Receiver Operating Characteristic curve (ROC), performance metrics (Accuracy/Sensitivity/Specificity/F1), and confusion matrix predicting "Response" vs. "Non-response" outcomes. (E)Visualization of the HPCs associated to tumor response. Description of histological features of each HPC are provided in Table S2.


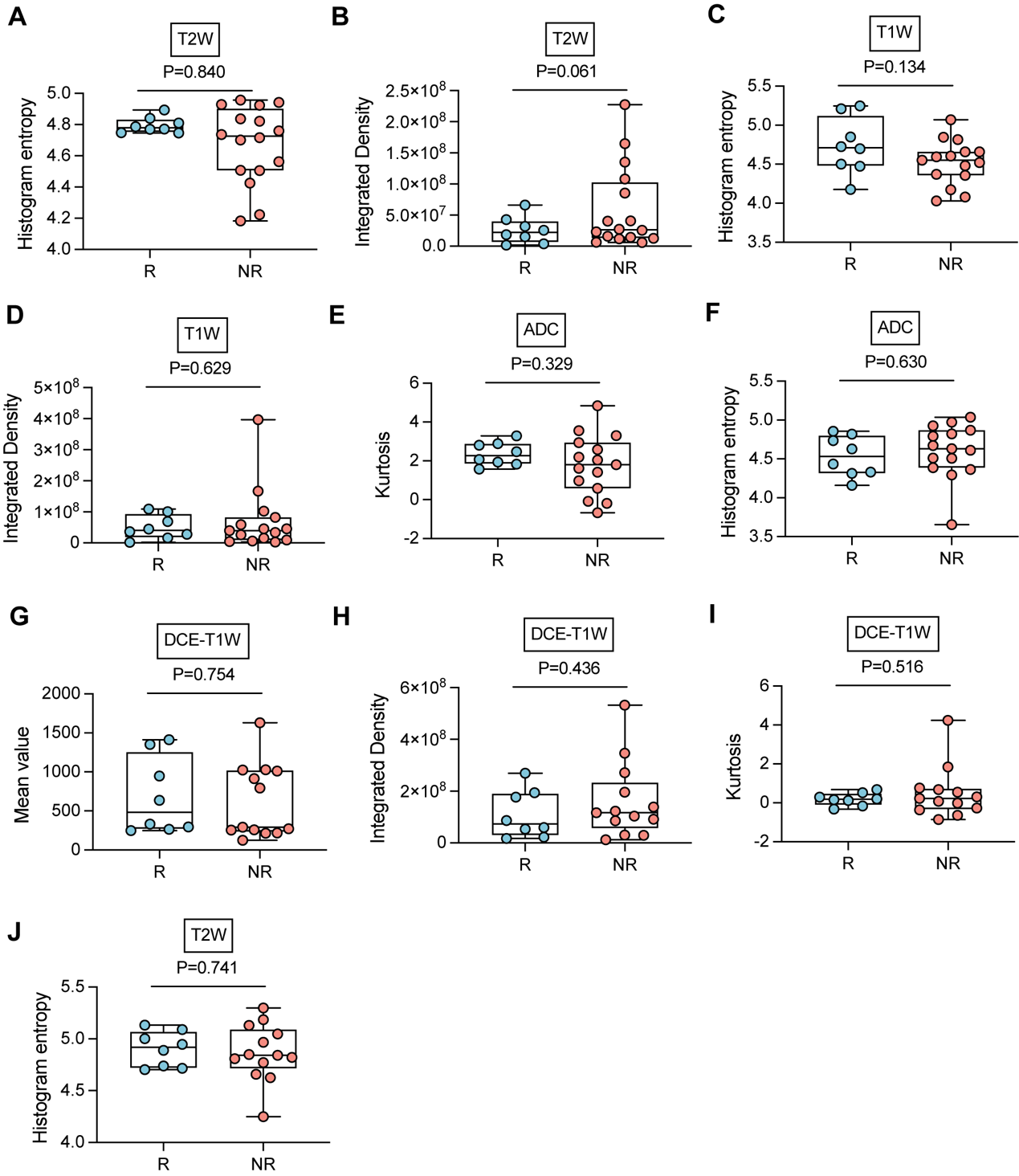


Figure S2. Box plot of additional baseline multiparametric MRI measurements between responders and non-responders.

This box plot presents the relationship between treatment response (Responders vs. Non-Responders) and the remaining baseline multiparametric MRI sequence parameters that were not included in Figure 3.The sequences and parameters displayed in the figure are as follows: T2W (integrated density and histogram entropy), T1W(kurtosis and mean intensity), ADC (kurtosis and histogram entropy), DCE-T1W(mean intensity, integrated density, kurtosis and histogram entropy).


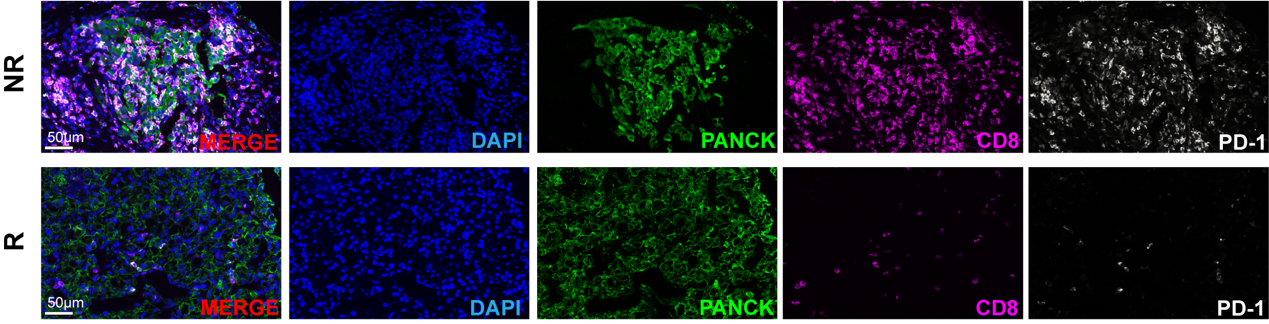


Figure S3. Representative images of the immunofluorescence analysis.

Representative immunofluorescence images show the difference in the proportion of CD8+ T cells within the tumor immune microenvironment between Responder and Non-Responder. Scale bar: 50 µm.


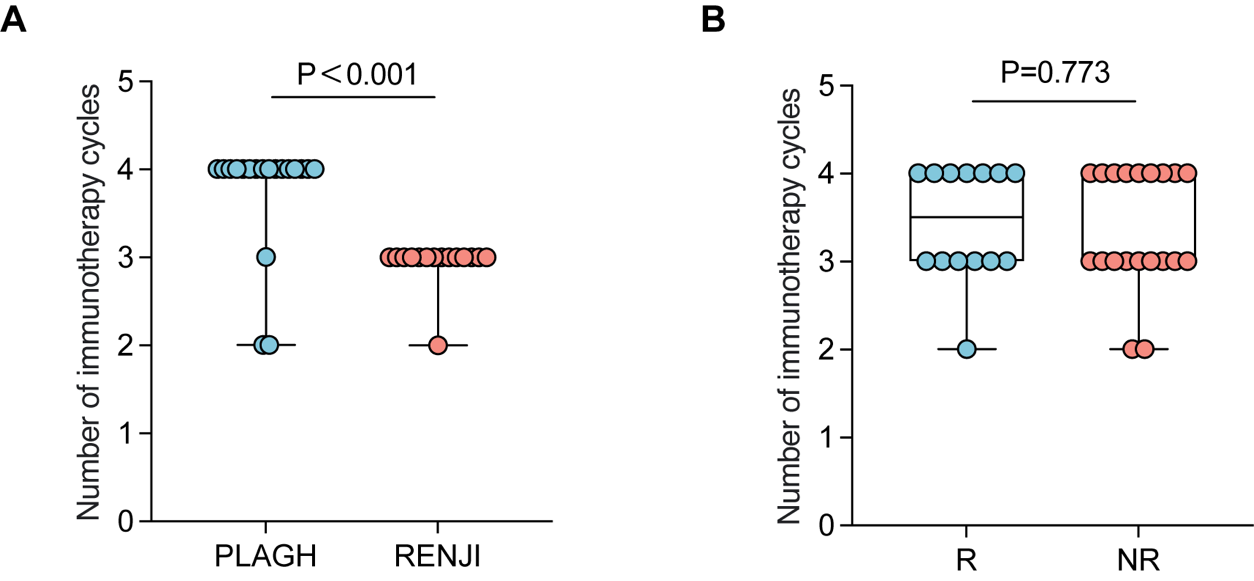


Figure S4. Comparison of immunotherapy cycles across different centers and their relationship with tumor response.

Abbreviation: R, Responder; NR, Non-responder.
